# Lanthanum Chloride in Vascular Calcification: Effects on Nano‐Hydroxyapatite and PPARγ/Wnt/β‐Catenin

**DOI:** 10.1096/fba.2025-00290

**Published:** 2026-05-27

**Authors:** Ting Zhang, Jialong Qian, Li Zhu, Zhihua Zhou, Xin Wang, Yan Liang, Luyu Wang, Chunsheng Zhang, Ren Bu, Hong Liu, Changjin Xu, Gang Li

**Affiliations:** ^1^ Department of Pharmacology College of Pharmacy, the Inner Mongolia Medical University, Jinshan Development Hohhot China; ^2^ Inner Mongolia University of Science and Technology Baotou Medical College Baotou China

**Keywords:** hydroxyapatite, PPARγ/Wnt/β‐catenin signaling pathway, vascular calcification

## Abstract

This study investigated the effects of lanthanum chloride on vascular calcification associated with chronic kidney disease (CKD) and the mechanisms involved in changes in nano‐hydroxyapatite. Vascular calcification was induced in CKD rats using a high‐phosphorus diet and adenine. Human vascular smooth muscle cells (hVSMCs) were calcified in vitro using sodium β‐glycerophosphate (β‐GP) and saturated nano‐hydroxyapatite. The effects of lanthanum chloride were evaluated using various analytical methods, including serum biochemistry, EVG, Vonkossa staining, Alizarin Red staining, Ca^2+^ detection, Western blotting, and transmission electron microscopy. The results showed that lanthanum chloride effectively inhibited calcium deposition and osteogenic differentiation, a finding confirmed in both in vivo and in vitro experiments. Scanning electron microscopy (SEM) analysis of the calcified crystals revealed altered crystal morphology and a decreased calcium‐to‐phosphorus ratio after treatment. X‐ray diffraction confirmed that these crystals were hydroxyapatite. Proteomic analysis indicated that the effects of lanthanum chloride were associated with apoptosis and the PPARγ/Wnt/β‐catenin signaling pathway. Lanthanum chloride inhibits apoptosis in human vascular smooth muscle cells (hVSMCs), while PPARγ inhibitors can reverse this effect. In summary, lanthanum chloride may significantly reduce vascular calcification in patients with CKD by altering the morphology of hydroxyapatite and activating PPARγ to inhibit apoptosis.

## Graphical abstract

This study elucidates the anti‐calcification mechanism of lanthanum chloride (LaCl₃) in rats with chronic kidney disease (CKD) and human vascular smooth muscle cells (hVSMCs). Unlike traditional phosphate binders, trace amounts of LaCl₃ can inhibit vascular calcification without lowering serum phosphorus levels. The mechanism involves LaCl₃ reducing calcification and apoptosis by altering the morphology and calcium‐to‐phosphorus ratio of nano‐hydroxyapatite crystals (physical interference) and by activating peroxisome proliferator‐activated receptor γ (PPARγ) to inhibit the Wnt/β‐catenin signaling pathway.

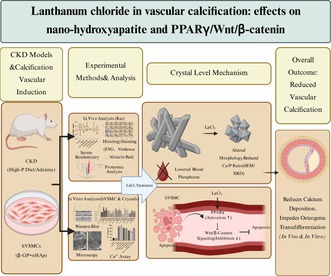

## 1 Introduction

Chronic kidney disease (CKD) is a significant global health burden, often progressing to end‐stage renal disease (ESRD). Cardiovascular disease, a complication of CKD, remains a leading cause of death in this population, primarily caused by vascular calcification (VC) [1, 2]. As a hallmark of CKD‐Mineral and Bone Disorder (CKD‐MBD), VC involves the pathological deposition of calcium‐phosphate minerals within the vasculature, a process significantly accelerated by hyperphosphatemia and uremic toxins [3, 4].

The pathogenesis of VC resembles active osteogenesis rather than passive precipitation. Hydroxyapatite (HAp) constitutes the primary mineral phase in calcified vessels. Under hyperphosphatemic conditions, vascular smooth muscle cells (VSMCs) secrete matrix vesicles that serve as nucleation sites for nano‐HAp crystals [5, 6]. These crystals are not inert; they actively modulate cellular behavior by promoting endocytosis, inflammation, and apoptosis [7, 8, 9]. Crucially, nano‐HAp accumulation drives VSMC transdifferentiation toward an osteoblast‐like phenotype—marked by the upregulation of Runx2 and BMP‐2—thereby creating a feed‐forward loop that exacerbates calcification [10, 11].

At the molecular level, this osteogenic reprogramming is governed by the interplay between the PPARγ and Wnt/β‐catenin signaling pathways. The mutual antagonism between PPARγ and the Wnt/β‐catenin pathway has been demonstrated to regulate the differentiation of mesenchymal stem cells (MSCs) into adipocytes and osteoblasts [12, 13, 14]. While Wnt/β‐catenin signaling is essential for embryonic development, its sustained activation in CKD drives renal fibrosis and vascular damage [15, 16, 17]. Conversely, PPARγ acts as a protective transcription factor, suppressing osteogenic differentiation. Research indicates a critical crosstalk mechanism: PPARγ agonists can preserve VSMC phenotype by inhibiting aberrant Wnt signaling (e.g., via sFRP2 upregulation) and promoting β‐catenin degradation [18, 19, 20]. In addition, pioglitazone, a PPARγ agonist, has been suggested to inhibit the canonical Wnt pathway, which attenuates VC. However, pro‐calcific stimuli often downregulate PPARγ, shifting the balance toward Wnt‐mediated osteogenesis [21].

Current clinical management of VC relies heavily on dietary restriction and oral phosphate binders; however, the therapeutic potential of intravenous lanthanum chloride remains under‐explored. Distinct from the mechanism of oral phosphate binders, this study investigates the direct effects of intravenous lanthanum chloride on VC. By simulating a high‐phosphorus environment to induce calcification, we aim to elucidate how lanthanum chloride modulates nano‐HAp morphology and regulates the underlying signaling networks to inhibit vascular calcification.

## 2 Materials and Methods

### 2.1 Reagents and Antibodies

Lanthanum chloride (≥ 99.9%, Sigma #298182), Lanthanum carbonate (API grade, Sigma #325767), Adenine (Sigma A2786, CAS 73–26‐5). RNA extraction: RNA Simple Total RNA Kit (Tiangen #DP429), Reverse transcription: ReverTra Ace qPCR RT Kit (Toyobo #FSQ‐101). Anti‐SM22α (Mouse mAb, SCBT sc‐53,932), Anti‐BMP‐2/4 (Mouse mAb, SCBT sc‐137,088), Anti‐Runx2 (Mouse mAb, SCBT sc‐101,135), Anti‐β‐Actin (Rabbit pAb, Solebao K101527P), Anti‐Lamin A (Rabbit pAb, Proteintech 10298‐1‐AP, RRID:AB_2296961), Anti‐Mouse IgG‐HRP (Abbkine A23910), Anti‐Rabbit IgG‐HRP (Abbkine A23920). Nuclear protein extraction: NE‐PER Kit (Thermo 78833), Protein quantification: BCA Assay Kit (Beyotime P032).

### 2.2 Animal Models and Interventions

Sixty male Wistar rats (6‐week‐old) were procured from Beijing Weitonglihua Experimental Animal Technology Co. Ltd. (Beijing; License: SCXK 2021‐0009). Animals were maintained under standard SPF conditions (23°C ± 1°C, 50% ± 5% humidity, 12‐h light/dark cycles) in the barrier facility of Inner Mongolia Medical University's Animal Center. Following a 7‐day acclimatization period with ad libitum access to food/water, experimental procedures were initiated. The research design, applied in our study, meets the Animals in Research: Reporting In Vivo Experiments (ARRIVE) guidelines and was also approved by the Medical Ethics Committee of Inner Mongolia Medical University (Approval No. YKD202201007). The experiment also complies with the guidelines of the European Parliament Directive 2010/63/EU on the protection of animals used for scientific purposes and the National Institutes of Health Guidelines for the Care and Use of Laboratory animals.

Adenine‐induced CKD model: Following a one‐week acclimatization period, rats were orally administered a daily gavage of 2% adenine suspension at a dose of 200 mg/kg from Weeks 1 to 2. Subsequently, the same concentration and dose were administered on alternate days from Weeks 3 to 4. Throughout Weeks 1 to 4, all rats were fed a diet containing 1.2% high‐phosphorus. In the control group, 10 rats with similar hair color and body size were selected and fed a standard SPF grade diet without any medications. The animals had ad libitum access to food and water. Following successful modeling, the CKD model rats were randomly assigned to the following groups: blank group, model group, 0.03 ng/kg LaCl_3_ group, 0.1 ng/kg LaCl_3_ group, 0.3 ng/kg LaCl_3_ group, and 0.3 g/kg La_2_(CO_3_)_3_ group (*n* = 10). Initially, 10 mice were randomly assigned to each experimental group to account for potential attrition. During the study, 3 rats from each group were excluded from the final analysis because they failed to meet the predefined criteria for successful disease modeling. Therefore, the final sample size included in all statistical analyses was 7 mice per group (*n* = 7). This sample size was sufficient to achieve statistical significance for our primary endpoint. CKD rats received the corresponding dose of LaCl_3_ solution via tail vein injection every 3 days for a duration of 8 weeks.

At the end of the experiment, Rat were anesthetized by 1.5%–2% isoflurane peripherally, and euthanized by intravenous injection of a lethal dose of pentobarbital sodium (50 mg/kg) through aortic tissue.

### 2.3 Serum Biochemical Analysis

At experimental endpoint, blood samples were collected from the abdominal aorta into anticoagulant‐free tubes and centrifuged at 3500 rpm for 15 min. The serum of the rats was taken and biochemical indexes such as creatinine, urea nitrogen, P, Ca^2+^, ALP, PTH and FGF23 in the serum were detected using the kit.

### 2.4 Elastica van Gieson (EVG) Dyeing

Paraffin sections underwent standard dewaxing and hydration. After staining with EVG solution A (5 min), slides were rinsed under running water. Differentiation was performed in twice‐diluted EVG solution B until elastic fibers appeared purplish‐black against a gray‐white background. Sections were counterstained in VG solution (1–3 min), rapidly washed, and dehydrated through three changes of absolute ethanol. Finally, slides were cleared in xylene I (30 s) and xylene II (5 min) before neutral balsam mounting for microscopic examination.

### 2.5 Von Kossa Staining

Paraffin sections were dewaxed and hydrated, then air‐dried. After application of Von Kossa stain, slides were ultraviolet‐irradiated for 4 h and rinsed in distilled water. Subsequent hematoxylin–eosin (HE) staining included:

1. Hematoxylin (5 min) → running water rinse
2. 1% acid ethanol differentiation (30 s) → running water rinse
3. 0.6% ammonia water bluing → running water rinse
4. Gradient dehydration: 85% ethanol (5 min) → 95% ethanol (5 min)
5. Eosin counterstain (5 min)

Sections were cleared in xylene and mounted with neutral balsam for microscopic analysis.

### 2.6 Western Blot

The expressions of osteoblastic phenotypic proteins, apoptosis‐related proteins, and pathway‐related proteins were detected. After the modeling was completed, the rat abdominal aorta was ground, the protein was extracted, the BCA kit was quantified in groups, and the sample amount was calculated. According to the molecular weight of the protein to be detected, the gel was prepared by SDS electrophoresis (80–150 V); the mold was turned at 400 mA current for 1 h; the room temperature was closed for 1 h; and the primary antibody was incubated overnight at 4°C. The next day, TBST rinsing (10 min/times, three times), secondary antibody (mouse resistance) incubation for 1 h, TBST rinsing (10 min/times, three times), and TBS rinsing once (10 min) for color determination.

### 2.7 Animal Immunofluorescence Co‐Localization

After the frozen slices were removed from the −80 refrigerator, they were placed at room temperature for 10–20 min, controlled by moisture, and fixed in 4% paraformaldehyde for 30 min. Wash in PBS buffer three times for 5 min each time. After the sections were slightly dried, circles were drawn around the tissues with a histochemical pen, and 0.5% TritonX‐100 (prepared with PBS) was permeable at room temperature for 20 min, and the sealing solution was closed at room temperature for 1 h. Gently shake off the sealing liquid, add the proportionally prepared primary antibody to the slices, and place the slices flat in a wet box at 4°C for overnight incubation. The slices were immersed in PBS buffer, and then washed three times on a shaker for 10 min each time. After the sections were slightly dried, fluorescent secondary antibody was added to cover the tissues and incubated at room temperature for 50 min away from light. The slices were immersed in PBS buffer and washed three times by shaking for 10 min each time. After drying, add DAPI dye solution in the circle and incubate at room temperature for 10 min away from light. The slices were placed in PBS buffer and washed three times with shaking for 10 min each time. After the slices were slightly dried, the slices were sealed with anti‐fluorescence quenching sealant, and nail polish was placed around the slide to bind the cover slide and the slide.

### 2.8 Cell Culture

The hVSMCs cell line was purchased from Shanghai Yubo Biotechnology Co. LTD. hVSMCs were cultured in a high‐sugar DMEM and 1% penicillin–streptomycin medium containing 10% fetal bovine serum, which is called a growth medium. After reaching cell fusion, cells from generations 5–7 were used in the experiment. Change the medium every 2 days. In order to induce cell calcification with phosphate, 10 mmol, β‐gp and nHAP were added to the medium for 14 days, respectively, and LaCl_3_ at different concentrations was added for 2 days on the 13th day.

### 2.9 DNA Damage

Appropriate amount of fixing solution was added to each well, fixed for 5–15 min, the fixing solution was sucked out, rinsed with washing solution (3 × 5 min), immunostaining blocking solution was added, closed at room temperature for 20 min, γ‐H2AX was added after suction, incubated at 4°C overnight, rinsed (3 × 10 min), anti‐rabbit 488 was added, rinsed (2 × 10 min). DAPI was added, stained at room temperature for 5 min, washed (3 × 5 min), appropriate amount of anti‐fluorescence quenching sealing liquid was added, and observed under confocal laser microscope.

### 2.10 Transmission Electron Microscopy

The cells of logarithmic growth stage were planted on a 6‐well plate with a density of 1 × 105 cells/well, with three pores per group. nHAP and β‐gp were incubated for 14 days, each well was treated with different concentrations of the drug for 48 h, the drug was discontinued, and 2.5% glutaraldehyde fixing solution was added at room temperature. After fixation for 5 min, the cells were gently scraped off with cell current in one direction, and the cell fluid was sucked into the centrifuge tube and placed into the centrifuge (3000 RPM, 2 min). The cell mass should be the size of a mung bean. After the fixative was discarded, a new electron microscope fixative was added, and the cells were lightly suspended in the fixative, fixed at room temperature for 30 min away from light, and then preserved at 4°C. After electron microscope vacuum debugging, the sliced sample was placed face up on the sample rack and sent into the mirror tube for observation. According to the characteristics of the film, the corresponding darkroom reagent was prepared and developed under the red light in the darkroom.

### 2.11 Flow Cytometry

After digestion with pancreatic enzyme without EDTA, the cells were collected by centrifugation (3000 r/min, 5 min) and washed with pre‐cooled PBS twice; each time, centrifugation was required (3000 r/min, 5 min). PBS was absorbed, the cells were re‐suspended with Binding Buffer, Annexin V‐FITC and PI dye were added, and the reaction was carried out at room temperature and away from light for 10–15 min. Finally, Binding Buffer was added, mixed, and placed on ice, and detected by flow cytometry within 1 h.

### 2.12 Alizarin Red Staining

VSMCs with logarithmic growth stage were selected for group plate culture, and the modeling and drug administration were completed. Then the original medium was discarded, fixed with 4% formaldehyde at 37°C for 30 min, washed 3 times with PBS, dyed with alizarin red solution at 37°C for 10 min, washed with PBS until no floating color, and images were captured under the microscope.

### 2.13 Von Kossa Staining

hVSMCs with logarithmic growth stage were selected for group plate culture, and the modeling and drug administration were completed and fixed with 4% formaldehyde at 37°C for 30 min, cleaned with PBS, stained with Von‐Kossa silver solution at room temperature for 10 min, exposed to UV for 30 min, cleaned with PBS, placed with Haibo solution at room temperature for 2 min, redyed with eosin dye for 5 min, cleaned with PBS without floating color, and images were captured under the microscope.

### 2.14 Calcium Quantification

To quantify in vitro calcium deposition, cells were treated under experimental conditions for 2 weeks. After cold PBS washes (×2), cells were lysed in 0.6 N HCl (4°C, 24 h). Calcium content was determined using a commercial assay kit (Cayman Chemical, USA) and normalized to total protein concentration.

### 2.15 Microscopic Morphology Analysis of Calcifiers

Nanocrystals were isolated from cell culture supernatants for analysis by X‐ray diffraction (XRD), scanning electron microscopy (SEM), and energy‐dispersive spectroscopy (EDS). Supernatants from β‐gp‐treated cells (cultured in 6‐well plates, with or without LaCl_3_ supplementation) were collected, purified, and centrifuged at 16,000 g for 1 h. The resulting pellets were washed with deionized water and centrifuged again. After drying, the crystal pellets were used for analysis.

For β‐gp‐treated cultures without La^3+^, one measurement represents crystals isolated from a total of 7 wells (each containing 2 mL culture medium). Due to reduced crystal formation in β‐gp cultures with La^3+^ supplementation, material from 21 wells (each containing 2 mL culture medium) was pooled per measurement to obtain sufficient quantities for XRD detection.

XRD analysis was performed using a PANalytical Empyrean diffractometer (PANalytical, Almelo, the Netherlands) in transmission mode. Samples were loaded into 0.5 mm soda glass capillaries (wall thickness: 0.01 mm). Measurements employed CuKα radiation generated by a fine‐focus sealed tube, with a focusing mirror and a PIXcel3D detector.

For combined SEM (GeminiSEM, Zeiss) and EDS elemental analysis (QUANTAX 200, Bruker), dried crystal pellets were mounted on copper tape and carbon‐coated. High‐resolution images were acquired using an Everhart‐Thornley SE detector. Accelerating voltages were 5 kV for morphological observation and 15 kV for micro‐elemental analysis.

### 2.16 Statistical Analysis

All statistical analyses were performed using SPSS. All statistical tests were two‐tailed, with a *p*‐value < 0.05 considered statistically significant. Based on data distribution, normally distributed continuous variables were expressed as mean ± standard deviation (Mean ± SD), non‐normally distributed continuous variables and ordinal variables were expressed as median (interquartile range), and categorical variables were expressed as scalars. Continuous variables were first assessed for normality using the Shapiro–Wilk test. If the data did not conform to a normal distribution, the Kruskal‐Wallis H test was used for intergroup comparisons, and Dunn's test (with Holm‐Bonferroni correction) was used for post hoc pairwise comparisons. If all data groups conform to a normal distribution, the Levene test is used to assess homogeneity of variance: if homogeneity of variance is found (*p* ≥ 0.05), one‐way ANOVA is used, followed by Tukey's HSD test for post hoc pairwise comparisons; if heterogeneity of variance is found (*p* < 0.05), Welch's ANOVA is used, followed by Games‐Howell's test for post hoc pairwise comparisons. All graphs were created using MetaLab software.

## 3 Results

### 3.1 Therapeutic Effect of Trace Lanthanum Ions on Adenine‐Induced Vascular Calcification in CKD Rats

**FIGURE 1:**
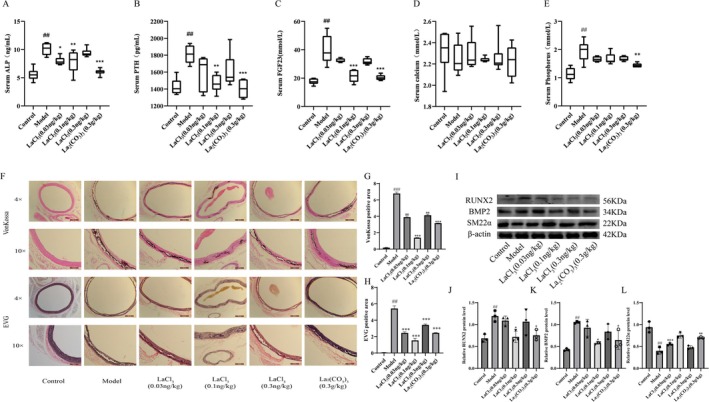
The therapeutic effect of trace lanthanum ions on adenine‐induced vascular calcification in rats with chronic kidney disease. (A) Serum alkaline phosphatase (ALP) (*n* = 7 per group). (B) Serum parathyroid hormone (PTH) (*n* = 7 per group). (C) Serum fibroblast growth factor 23 (FGF23) (*n* = 7 per group). (D) Serum calcium (*n* = 7 per group). (E) Serum phosphorus (*n* = 7 per group). (F–H) Von Kossa staining and EVG staining (*n* = 3 per group). Scale bar, 200 μm. (I–L) Western blot analysis and quantification of osteogenic phenotype markers (RUNX2, BMP2) and contractile phenotype marker (SM22α) (*n* = 3 per group). Statistical significance was assessed using one‐way ANOVA. All values are presented as means ± SEM, *n* = 7 per group. Compared with the Control group,^*##*^ *p* < 0.01; Compared with Model group, *p < 0.05, **p < 0.01,***p < 0.001; Compared with Lanthanum chloride group, ^&^ *p* < 0.05, ^&&^ *p* < 0.01.

Serum biochemical analysis of rats with vascular calcification due to chronic kidney disease (CKD) after 8 weeks of drug administration showed that, compared with the model group, lanthanum chloride significantly reduced the levels of alkaline phosphatase (ALP) (Figure 1A), parathyroid hormone (PTH) (Figure 1B), and fibroblast growth factor 23 (FGF23) (Figure 1C) (*p* < 0.05), with the medium‐dose group showing the best effect. However, lanthanum chloride had no significant effect on serum phosphorus levels (Figure 1D,E). EVG and Von Kossa staining results showed that after drug administration, elastic fibers in blood vessels were broken, and black calcium deposits appeared in the vascular media, indicating a decrease in the degree of calcification (Figure 1F–H). Western blot results showed that tail vein injection of a small amount of lanthanum chloride significantly increased the expression of SM22α protein and decreased the expression of BMP2 and RUNX2 proteins (Figure 1I–L). These results indicate that lanthanum chloride can treat adenine‐induced vascular calcification in rats with CKD.

Unlike traditional oral phosphate‐lowering drug treatment strategies, lanthanum chloride has no phosphate‐lowering effect, suggesting that lanthanum chloride may inhibit vascular calcification through a novel mechanism that may be related to changes in the crystal type, composition, and physical state of the mineral.

### 3.2 Lanthanum Ions Play a Role in the Treatment of Vascular Calcification Through Nano‐Hydroxyapatite

**FIGURE 2:**
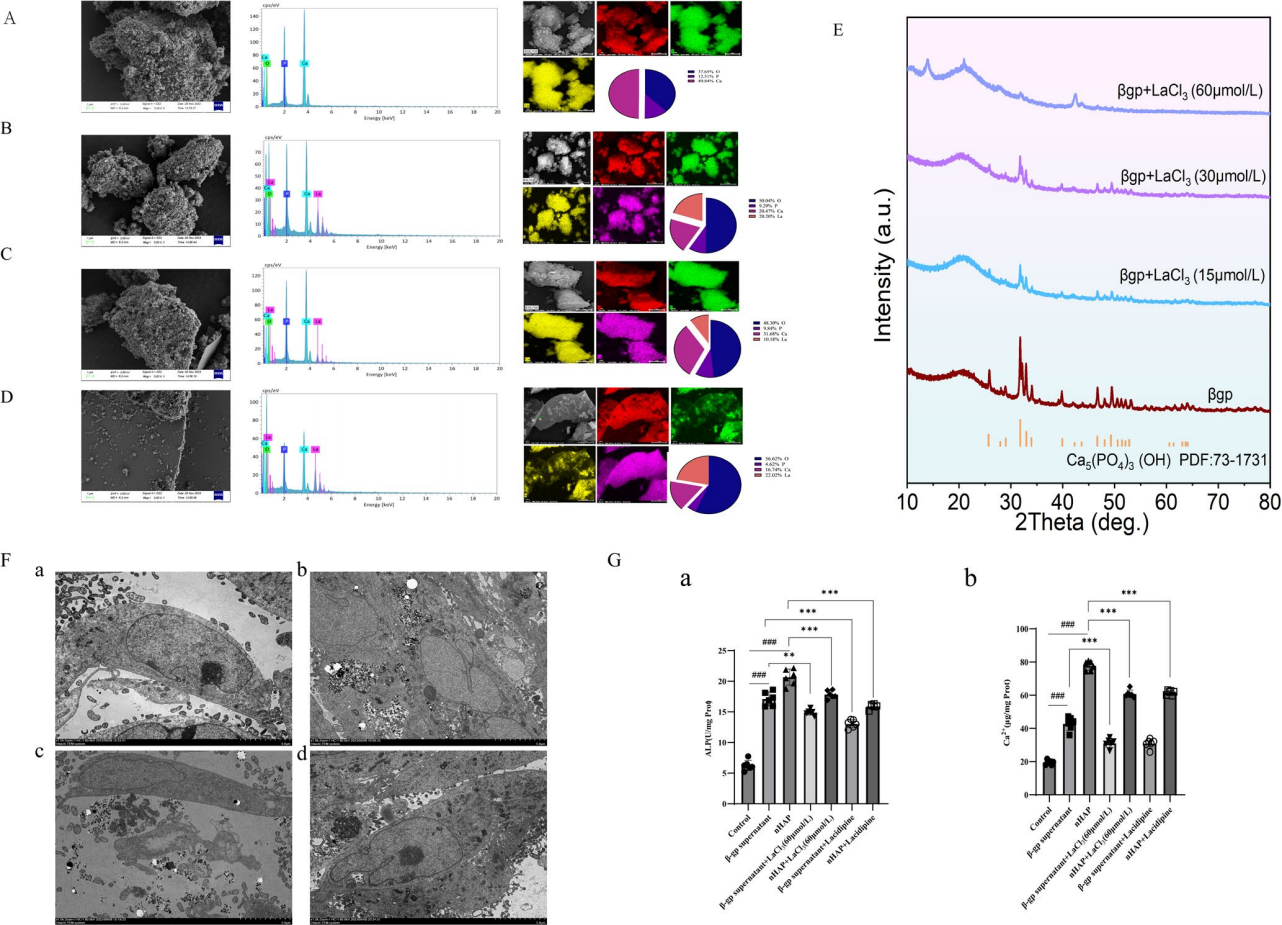
Lanthanum ions play a role in the treatment of vascular calcification via nano‐hydroxyapatite. (A–D) Scanning electron microscopy, energy dispersive spectroscopy, and X‐ray diffraction results of the β‐gp group, β‐gp + La‐L group, β‐gp + La‐M group, and β‐gp + La‐H group. (E) XRD results. (F) Transmission electron microscopy. (a) Control group. (b) Model group. (c) Lanthanum chloride group (0.1 ng/kg). (d) Lanthanum carbonate group (*n* = 3 per group). (G) Calcification induced by β‐gp treatment of hVSMCs, followed by treatment of hVSMCs with supernatant, (a) ALP. (b) Ca^2+^. (*n* = 6 per group) (Statistical significance was assessed using one‐way ANOVA. All values are presented as means ± SEM, *n* = 3 per group. Compared with the Control group, ^*##*^ *p* < 0.01; Compared with Model group, *p < 0.05, **p < 0.01,***p < 0.001; Compared with Lanthanum chloride group, ^&^ *p* < 0.05, ^&&^ *p* < 0.01).

To observe the mineralogical characteristics of calcifications and investigate the mechanism by which lanthanum chloride inhibits vascular calcification based on mineral morphology, this study employed X‐ray diffraction, scanning electron microscopy, and spectral analysis to analyze the in vitro calcification deposition induced by β‐gp and the morphology and elemental composition of the calcifications after drug administration. Scanning electron microscopy results showed that the morphology of the calcification crystals in the lanthanum chloride group changed compared to the model group, and the ratio of Ca to P decreased after the addition of lanthanum chloride. X‐ray diffraction analysis comparing the diffraction peaks of the calcification particles revealed that the diffraction peaks of the mineral crystals were consistent with the crystal plane diffraction peaks of the hydroxyapatite standard card (JCPDS 73–1731), confirming that the mineral crystals were hydroxyapatite (Figure 2A–E). Transmission electron microscopy (TEM) results showed that lanthanum chloride significantly inhibited the increase of calcification crystals in human vascular smooth muscle cells (hVSMCs) induced by nano‐hydroxyapatite (nHAP) (Figure 2F). Treatment of hVSMCs with calcified supernatant revealed a similar calcification‐inducing effect to nHAP, and lanthanum chloride significantly reduced the degree of calcification (Figure 2G). Western blotting results showed that lanthanum chloride significantly inhibited the expression levels of Runx2 and Bmp2 proteins induced by nHAP and significantly reduced the expression level of SM22α protein (Figure 6A).

These results indicate that lanthanum ions play a role in the treatment of vascular calcification through nano‐hydroxyapatite, which may be related to the alteration of the crystal type of nano‐hydroxyapatite.

### 3.3 Inhibitory Effect of Lanthanum Ion on Vascular Calcification In Vitro

To mimic the biological process of vascular calcification, we simultaneously treated hVSMCs with β‐gp in vitro.

**FIGURE 3:**
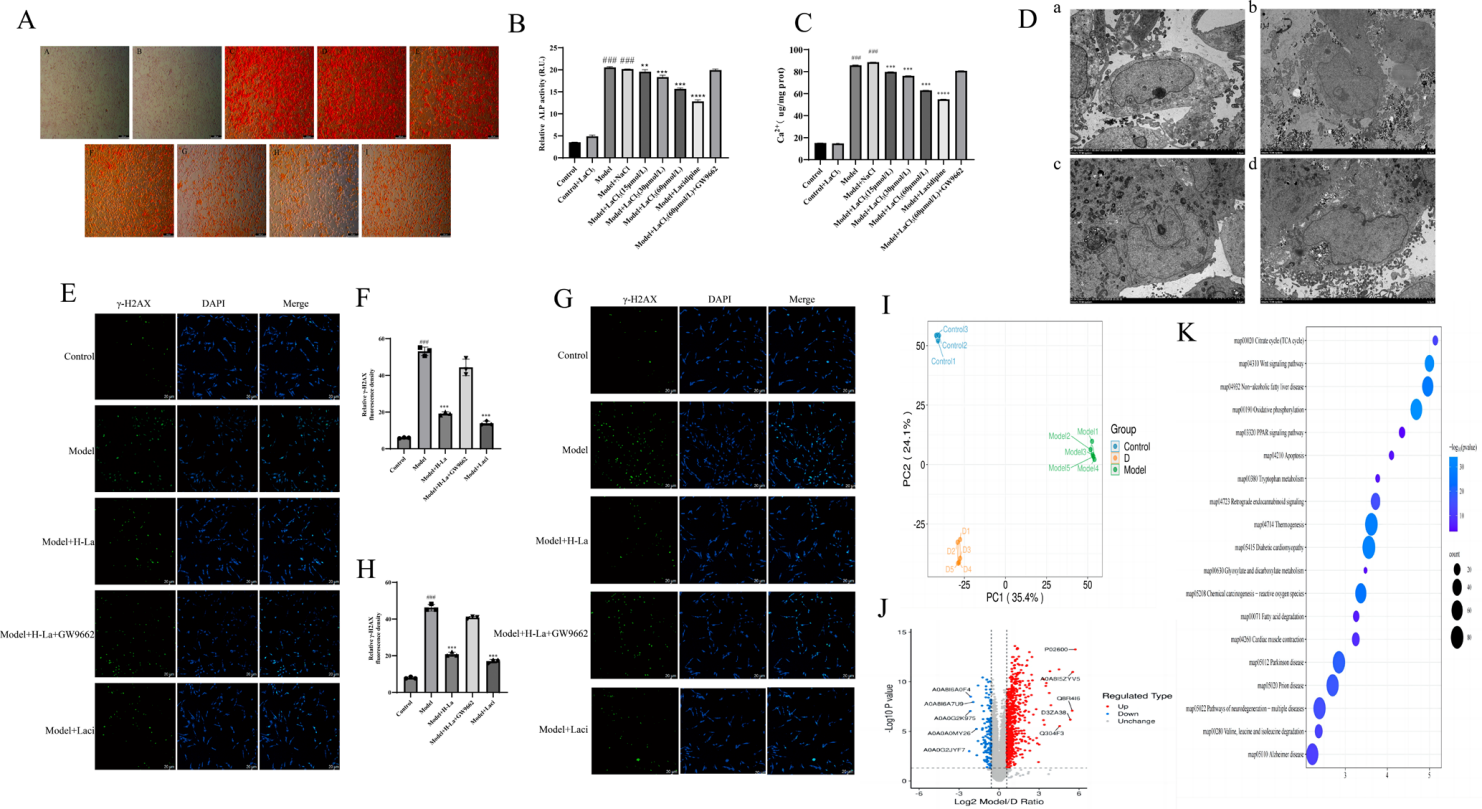
Inhibitory effect of lanthanum ions on in vitro vascular calcification. (A) Alizarin Red S staining. (a‐A) Control group. (a‐B) Control group + lanthanum chloride (60 μM) group. (a‐C) Model group. (a‐D) Model group + sodium chloride group. (a‐E) Model group + lanthanum chloride (15 μM) group. (a‐F) Model group + lanthanum chloride (30 μM) group. (a‐G) Model group + lanthanum chloride (60 μM) group. (a‐H) Model group + lacidipine group. (a‐I) Model group + lanthanum chloride (60 μM) + GW9662 group. (B, C) ALP and Ca^2+^ levels. (D) Transmission electron microscopy results. (a) Control group. (b) Model group. (c) Lanthanum chloride group (0.1 ng/kg). (d) Lanthanum carbonate group (*n* = 3 per group). (E, F) β‐gpDNA damage detection. (G, H) nHAP DNA damage detection. (*n* = 3 per group). (I–K) Proteomics. (*n* = 3 per group). (D) Proteomic. (Statistical significance was assessed using one‐way ANOVA. All values are presented as means ± SEM, *n* = 3 per group. Compared with the Control group, ^*##*^ *p* < 0.01; Compared with Model group, *p < 0.05, **p < 0.01,***p < 0.001; Compared with Lanthanum chloride group, ^&^ *p* < 0.05, ^&&^ *p* < 0.01).

Alizarin Red staining results showed that lanthanum chloride reduced β‐gp‐induced calcium deposition in a dose‐dependent manner. Results regarding calcium content and ALP activity were consistent with this (Figure 3A–C). Western blotting also showed that lanthanum chloride significantly inhibited β‐gp‐induced Runx2 and Bmp2 protein expression levels and significantly reduced SM22α protein expression levels (Figure 6B). Transmission electron microscopy results showed that lanthanum chloride significantly inhibited the increase of β‐gp‐induced calcification crystals in hVSMCs (Figure 3D). DNA damage results showed that lanthanum chloride significantly reduced the fluorescence intensity of β‐gp and nHAp‐induced γ‐H2AX. Lanthanum chloride reduced DNA damage (Figure 3E–H). Proteomics results suggest that lanthanum chloride treatment of vascular calcification may be related to apoptosis and the PPARγ/Wnt/β‐catenin signaling pathway (Figure 3I–K).

### 3.4 Lanthanum Ions Inhibit Vascular Calcification by Inhibiting the Apoptosis of Vascular Smooth Muscle Cells Induced by nHAP

**FIGURE 4:**
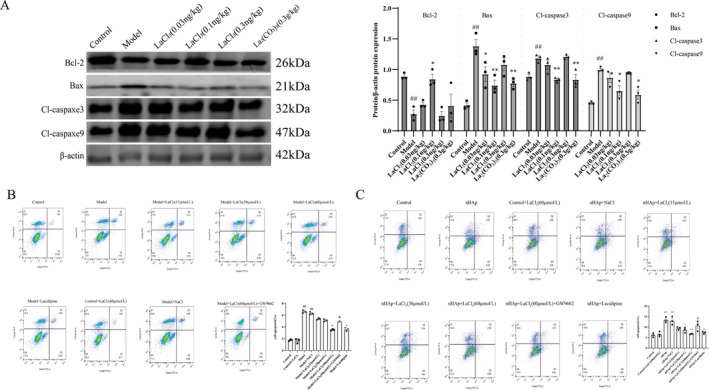
Lanthanum ions inhibit vascular calcification by suppressing nHAp‐induced apoptosis in vascular smooth muscle cells. (A) Western blot analysis and quantitative analysis of apoptosis markers (Bcl‐2, Bax, Cl‐caspase3, Cl‐caspase9) (*n* = 3 per group). (B) Flow cytometry detection of β‐gp‐induced apoptosis (*n* = 3 per group). (C) Flow cytometry detection of nHAp‐induced apoptosis (*n* = 3 per group). Statistical significance was assessed using one‐way ANOVA. All values are presented as means ± SEM, *n* = 3 per group. Compared with the Control group, ^*##*^ *p* < 0.01; Compared with Model group, *p < 0.05, **p < 0.01, ***p < 0.001; Compared with Lanthanum chloride group, ^&^ *p* < 0.05, ^&&^ *p* < 0.01.

Western blot analysis showed that lanthanum chloride and lacidipine significantly downregulated the expression of Bax and Caspase‐3/Cleaved Caspase‐3 in the aorta of CKD rats, while upregulating Bcl‐2 protein levels (*p* < 0.05; Figure 4A). Flow cytometry results showed that the addition of lanthanum chloride and lacidipine significantly reduced the apoptosis rate of β‐gp and HAP‐induced hVSMCs (Figure 4B,C). These results indicate that lanthanum ions inhibit vascular calcification by suppressing nHAP‐induced vascular smooth muscle cell apoptosis.

### 3.5 Lanthanum Ions Inhibit Vascular Calcification by Inhibiting the nHAp‐Activated PPARγ/Wnt/β‐Catenin Signaling Pathway

**FIGURE 5:**
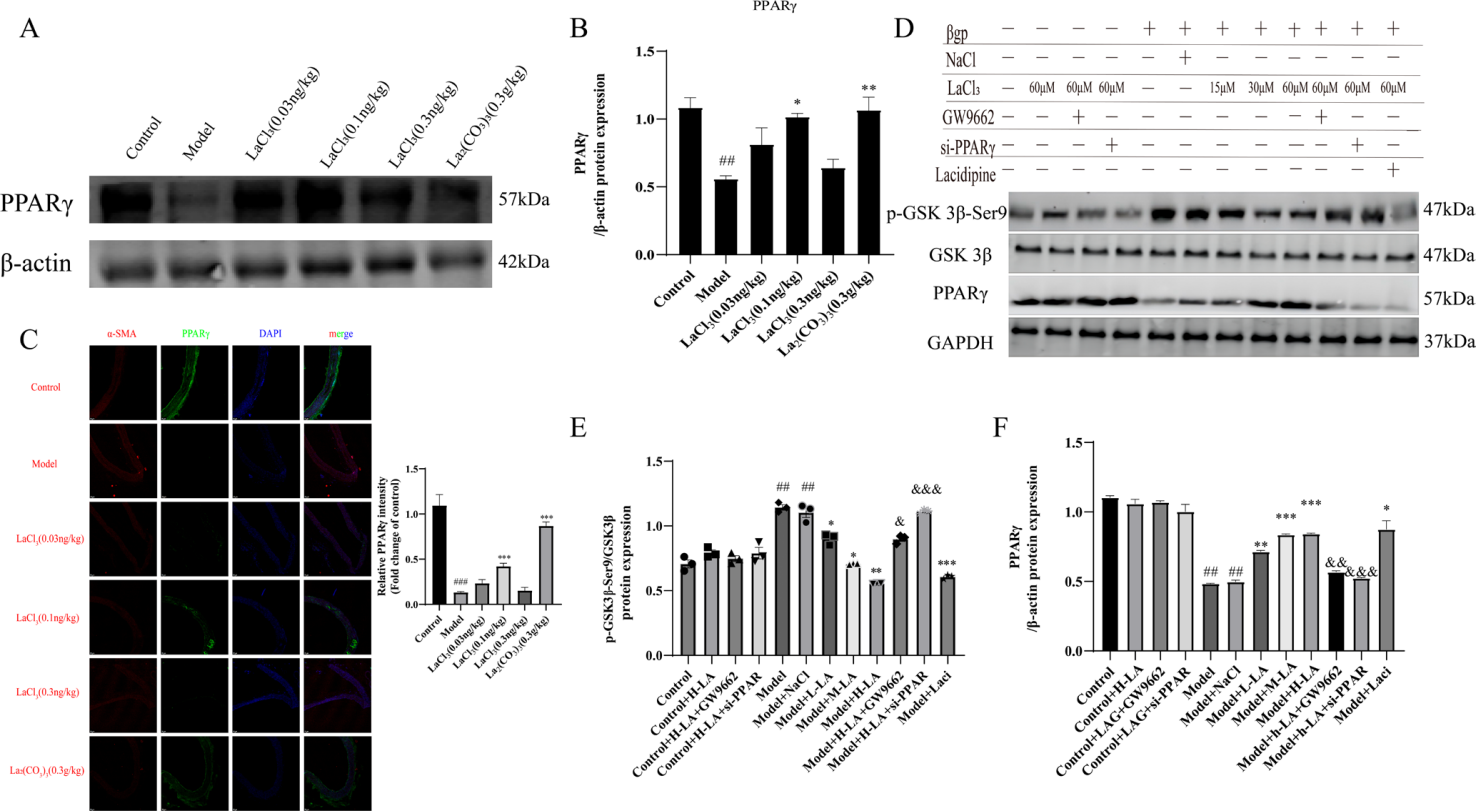
Lanthanum ions inhibit vascular calcification by suppressing the nHAp‐activated PPARγ/Wnt/β‐catenin signaling pathway. (A, B) Western blot analysis and quantification of PPARγ (*n* = 3 per group). (C) Aortic immunofluorescence co‐localization assay and quantification (*n* = 3 per group). (D–F) Western blot analysis and quantification of p‐GSK3β‐Ser9/GSK3β and PPARγ (*n* = 3 per group). (Statistical significance was assessed using one‐way ANOVA. All values are presented as means ± SEM, *n* = 3 per group. Compared with the Control group, ^*##*^ *p* < 0.01; Compared with Model group, *p < 0.05, **p < 0.01, ***p < 0.001; Compared with Lanthanum chloride group, ^&^ *p* < 0.05, ^&&^ *p* < 0.01, ^&&&^ *p* < 0.001).

Western blot results showed that lanthanum chloride could reverse the decrease in PPARγ protein expression in calcified CKD rats (Figure 5A,B). Immunofluorescence co‐localization experiments showed that the fluorescence intensity of PPARγ was decreased in the model group, but returned to normal after lanthanum ion treatment (Figure 5C). In vitro experiments showed that lanthanum ions could significantly reduce p‐GSK3β‐Ser9/GSK3β protein levels and significantly increase PPARγ levels (Figure 5D–F). These results indicate that lanthanum chloride inhibits vascular calcification by suppressing the PPARγ/Wnt/β‐catenin signaling pathway.

### 3.6 Lanthanum Chloride Inhibits Osteogenic Transdifferentiation and Apoptosis of Vascular Smooth Muscle Cells Through PPARγ/Wnt/β‐Catenin Signaling Pathway

**FIGURE 6:**
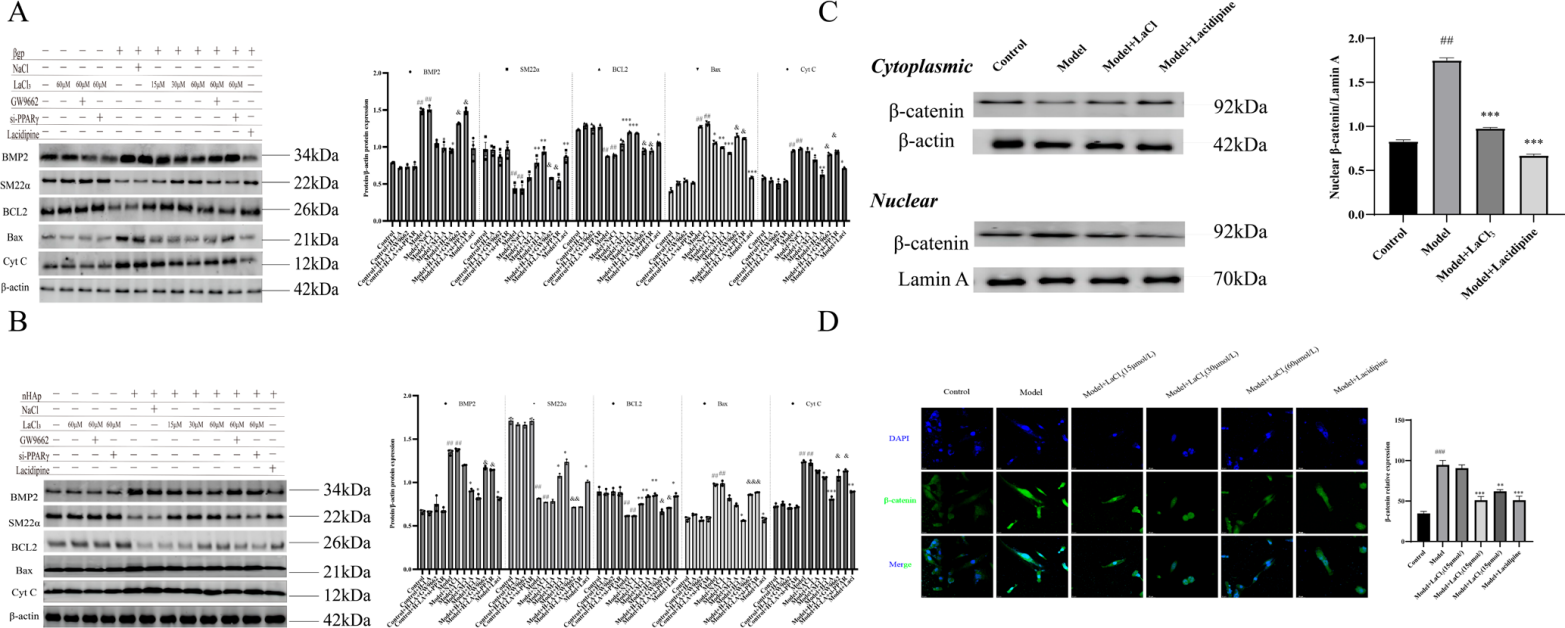
Lanthanum chloride inhibits osteogenic transdifferentiation and apoptosis of vascular smooth muscle cells via the PPARγ/Wnt/β‐catenin signaling pathway. (A) Western blot analysis and quantification of osteogenic transdifferentiation and apoptosis markers (BMP2, SM22α, Bcl‐2, Bax, Cyt C) (β‐gp, *n* = 3 per group). (B) Western blot analysis and quantification of osteogenic transdifferentiation and apoptosis markers (BMP2, SM22α, Bcl‐2, Bax, Cyt C) (nHAp, *n* = 3 per group). (C) Western blot analysis and quantification of β‐catenin in cytoplasm and nucleus (*n* = 3 per group). (D) Immunofluorescence and quantification (*n* = 3 per group). (Statistical significance was assessed using one‐way ANOVA. All values are presented as means ± SEM, *n* = 3 per group. Compared with the Control group, ^*##*^ *p* < 0.01; Compared with Model group, *p < 0.05, **p < 0.01,***p < 0.001; Compared with Lanthanum chloride group, ^&^ *p* < 0.05, ^&&^ *p* < 0.01, ^&&&^ *p* < 0.001).

In vitro experiments showed that treatment with β‐gp and nHAp increased osteogenic transdifferentiation and apoptosis in hVSMCs, while treatment with lanthanum chloride significantly reduced calcification and apoptosis levels (Figure 6A,B). Simultaneously, β‐gp treatment increased nuclear translocation of β‐catenin, while lanthanum chloride inhibited nuclear translocation of β‐catenin (Figure 6C,D).

## 4 Conclusion

This study found that lanthanum chloride can affect calcium deposition, phenotypic transformation, and apoptosis in adenine‐induced chronic kidney disease (CKD) rats and human vascular smooth muscle cells (hVSMCs), and that lanthanum chloride may exert its anti‐calcification effect by altering crystal structure and activating PPARγ to inhibit the Wnt/β‐catenin signaling pathway. This provides new insights into the therapeutic mechanism of vascular calcification. VC refers to the pathological deposition of calcium and phosphate minerals in the arterial wall, which is very common in CKD patients. Vascular calcification increases the risk of cardiovascular events in CKD patients. Moderate calcification is the most prominent pathological feature of vascular calcification in CKD patients [22]. It is mainly regulated by vascular smooth muscle cells. CKD‐related vascular calcification was initially thought to be a passive process of calcium phosphate deposition, but it is now generally believed that its pathogenesis is an actively regulated, complex, cell‐mediated process with many similarities to bone formation [23]. Clinically used drugs for the treatment of vascular calcification mainly include: calcium phosphate binders, polyphosphate binders, lanthanum carbonate, magnesium phosphate binders, and iron phosphate binders [24]. This study found that lanthanum chloride can reduce aortic calcification, ALP activity, and PTH and FGF23 levels in CKD rats. In vitro experiments also confirmed that lanthanum ions can reduce nHAp and β‐gp‐induced hVSMC calcification. These results further deepen our understanding of the protective effect of lanthanum chloride against CKD‐induced vascular calcification. Surprisingly, lanthanum chloride had no significant effect on serum phosphorus, a phenomenon that requires further investigation through subsequent experiments. Specifically, we provide clear physicochemical evidence to demonstrate this direct alteration of crystal structure. TEM, SEM, and XRD studies show that the addition of La^3+^ fundamentally changes the crystal morphology, particle size distribution, and lattice structure of nHAp. This structural change stems from the direct interaction between La^3+^ and the apatite crystallization process. This key finding closely links the physical modification of the crystal microstructure by La^3+^ to its macroscopic biological effects. The generally accepted view among scholars is that the transition of VSMCs from a contractile phenotype to a state similar to osteoblast differentiation is a key stage in the pathological calcification cascade. Under environmental pressures favorable to calcification progression, these VSMCs begin to undergo phenotypic changes, characterized by a decrease in contractile markers, along with reductions in SM22 and α‐SMA. Observations show increased transcription levels of genes related to bone development, particularly RUNX2, BMP, OPN, osteocalcin, and ALP. RUNX2, as a typical transcription factor, plays a role in the normal osteoblast differentiation pathway and is widely considered a key factor in regulating osteogenic transformation and subsequent mineralization of VSMCs [25], further highlighting the importance of the aforementioned transition. Confirmatory evidence from controlled laboratory environments and living organisms supports our view: lanthanum chloride can effectively inhibit the increase of RUNX2, indicating that it can alleviate the tendency of VSMCs to reprogram to osteogenic reprogramming. Proteomics studies suggest that the mechanism by which lanthanum chloride inhibits vascular calcification may be closely related to regulatory pathways such as the PPARγ/Wnt/β‐catenin signaling pathway. Canonical Wnt signaling pathway‐mediated signal transduction stabilizes β‐catenin in the cytoplasm and promotes its translocation to the nucleus. Nuclear β‐catenin then forms a complex with the T‐cell cytokine/lymphocyte‐enhancing factor (TCF/LEF) family, driving transcription of genes regulating cell fate, including osteogenic differentiation of mesenchymal stem cells. The Wnt/β‐catenin pathway is a major regulator of bone formation signaling and promotes VC by directly targeting RUNX2 and PiT‐1 [26]. Our results indicate that lanthanum chloride inhibits activation of the Wnt/β‐catenin pathway and impedes nuclear translocation of β‐catenin. Significant interactions between PPARγ and the Wnt/β‐catenin signaling pathway have been elucidated. PPARγ plays a complex role in regulating the dynamics of epithelial‐to‐mesenchymal phenotype transition in oncogenic mesenchymal cells and adipose‐derived fibroblasts in adipose tissue.

A significant interaction exists between PPARγ and the Wnt/β‐catenin signaling pathway. PPARγ plays a complex role in regulating the dynamic transition of epithelial to mesenchymal phenotype in oncogenic cells and adipose‐derived fibroblasts in the alveolar matrix [27, 28]. In this study, we found significantly reduced PPARγ levels in the aortic structures of rodents with CKD, while lanthanum ion treatment increased PPARγ levels. Conversely, lanthanum ion treatment effectively inhibited β‐catenin overexpression. Experimental data from calcified VSMCs showed that lanthanum chloride inhibited both the activation of the Wnt/β‐catenin signaling pathway and nuclear transport of β‐catenin, suggesting that PPARγ may be activated while lanthanum chloride simultaneously inhibits the Wnt/β‐catenin signaling pathway. To demonstrate that PPARγ is not a bystander in this process but a key mediator connecting crystallization and vascular protection, we employed a loss‐of‐function strategy for further validation. Using two independent approaches—gene knockdown (si‐PPARγ) and pharmacological antagonism (GW9662)—we consistently found that once PPARγ function was blocked, the reversal effect of LaCl_3_ on nHAP‐induced β‐catenin accumulation and SM22‐α downregulation was almost completely lost. The reverse verification results strongly suggest that PPARγ activation may be a factor in the anti‐calcification effect of LaCl_3_.

It is important to note that the pharmacological strategy used in this study differs significantly from the clinical application of lanthanum carbonate (La_2_(CO_3_)_3_). Clinically (La_2_(CO_3_)_3_), as a non‐calcium phosphate binder, has extremely low systemic absorption (< 0.002%) and is designed to reduce serum phosphate levels. In contrast, this study used intravenous injection of soluble lanthanum chloride (LaCl_3_) to achieve direct vascular targeting. Oral therapy relies on passive, inefficient absorption and suffers from a significant first‐pass effect, while our intravenous injection strategy ensures precise delivery of trace amounts of LaCl_3_ (0.3 ng/kg) into the bloodstream. Therefore, our results suggest a novel treatment approach—systemic micro‐lanthanum therapy—rather than indicating that current oral regimens directly protect blood vessels. This distinction allows for potent anti‐calcification at doses far below the systemic burden that high‐dose oral accumulation might cause.

**FIGURE 7:**
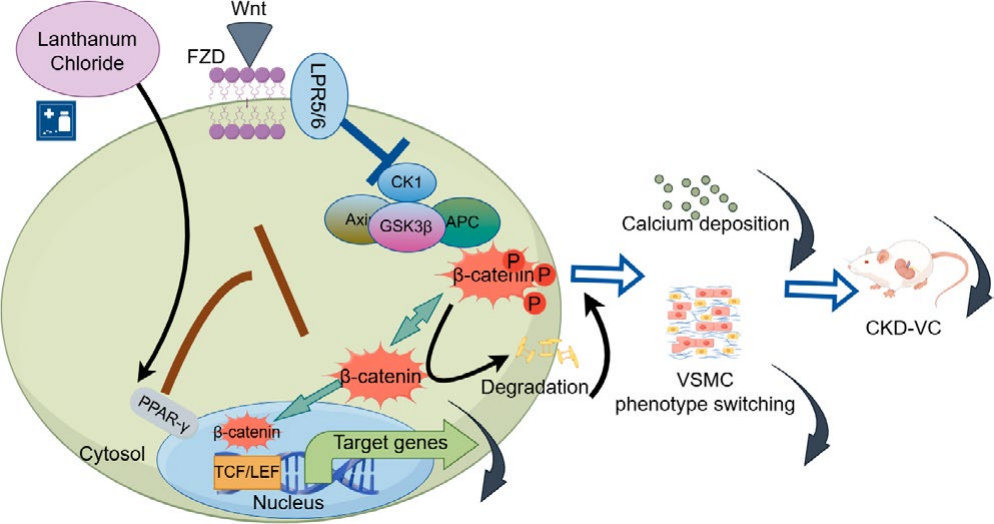
This is the pathway and mechanism diagram involved in this article. The figure illustrates CKD‐VC: Wnt ligands activate the Wnt/β‐catenin pathway via FZD and LPR5/6, stabilizing β‐catenin to trigger target gene transcription in the nucleus, driving VSMC phenotypic switching and calcium deposition. Lanthanum chloride regulates β‐catenin via cytoplasmic PPARγ to interfere with this process.

In summary, we found that lanthanum chloride reduces calcium deposition, osteogenic transdifferentiation, and apoptosis in hVSMCs in an adenine‐induced CKD rat model (Figure 7).

## Author Contributions

Gang Li contributed to the study conception and design. Ting Zhang and Jialong Qian contributed to the data analysis and the writing of the first draft of the manuscript. Li Zhu, Zhihua Zhou, Xin Wang, and Yan Liang contributed to the material preparation work. Luyu Wang and Chunsheng Zhang contributed to performing the cell and animal experiments. Ren Bu, Hong Liu, and Changjin Xu contributed to the data management of this experiment.

## Funding

This research was funded by the Science and Technology Department of Inner Mongolia Autonomous Region (2022JQ01), the Major Science and Technology Project of Inner Mongolia Autonomous Region (zdzx201805), and the Innovation Center for Ethnic Medicine of Inner Mongolia Medical University.

## Conflicts of Interest

The authors declare no conflicts of interest.

## Data Availability

The data underlying this article will be shared on reasonable request to the corresponding author.

## References

[1] B. Bikbov , C. A. Purcell , A. S. Levey , et al., “Global, Regional, and National Burden of Chronic Kidney Disease, 1990–2017: A Systematic Analysis for the Global Burden of Disease Study 2017,” Lancet 395 (2020): 709–733. 32061315 10.1016/S0140-6736(20)30045-3 PMC7049905

[2] J. Jankowski , J. Floege , D. Fliser , M. Böhm , and N. Marx , “Cardiovascular Disease in Chronic Kidney Disease: Pathophysiological Insights and Therapeutic Options,” Circulation 143 (2021): 1157–1172. 33720773 10.1161/CIRCULATIONAHA.120.050686 PMC7969169

[3] KDIGO , “KDIGO Clinical Practice Guideline for the Diagnosis, Evaluation, Prevention, and Treatment of Chronic Kidney Disease‐Mineral and Bone Disorder (CKD‐MBD),” Kidney International 113 (2009): S1–S130. 10.1038/ki.2009.188 19644521

[4] M. Wu , C. Rementer , and C. M. Giachelli , “Vascular Calcification: An Update on Mechanisms and Challenges in Treatment,” Calcified Tissue International 93 (2013): 365–373. 23456027 10.1007/s00223-013-9712-z PMC3714357

[5] S. W. Ha , H. L. Jang , K. T. Nam , and G. R. Beck, Jr. , “Nano‐Hydroxyapatite Modulates Osteoblast Lineage Commitment by Stimulation of DNA Methylation and Regulation of Gene Expression,” Biomaterials 65 (2015): 32–42. 26141836 10.1016/j.biomaterials.2015.06.039 PMC4508253

[6] D. Proudfoot , J. N. Skepper , L. Hegyi , M. R. Bennett , C. M. Shanahan , and P. L. Weissberg , “Apoptosis Regulates Human Vascular Calcification In Vitro: Evidence for Initiation of Vascular Calcification by Apoptotic Bodies,” Circulation Research 87 (2000): 1055–1062. 11090552 10.1161/01.res.87.11.1055

[7] Y. Sun , X. R. Zeng , L. Wenger , and H. S. Cheung , “Basic Calcium Phosphate Crystals Stimulate the Endocytotic Activity of Cells—Inhibition by Anti‐Calcification Agents,” Biochemical and Biophysical Research Communications 312 (2003): 1053–1059. 14651978 10.1016/j.bbrc.2003.11.048

[8] M. P. Morgan and G. M. McCarthy , “Signaling Mechanisms Involved in Crystal‐Induced Tissue Damage,” Current Opinion in Rheumatology 14 (2002): 292–297. 11981329 10.1097/00002281-200205000-00017

[9] I. Nadra , J. C. Mason , P. Philippidis , et al., “Proinflammatory Activation of Macrophages by Basic Calcium Phosphate Crystals via Protein Kinase C and MAP Kinase Pathways: A Vicious Cycle of Inflammation and Arterial Calcification?,” Circulation Research 96 (2005): 1248–1256. 15905460 10.1161/01.RES.0000171451.88616.c2

[10] L. H. Huang , H. Liu , J. Y. Chen , et al., “Seaweed *Porphyra yezoensis* Polysaccharides With Different Molecular Weights Inhibit Hydroxyapatite Damage and Osteoblast Differentiation of A7R5 Cells,” Food & Function 11 (2020): 3393–3409. 32232300 10.1039/c9fo01732a

[11] M. Tsujihata , “Mechanism of Calcium Oxalate Renal Stone Formation and Renal Tubular Cell Injury,” International Journal of Urology 15 (2008): 115–120. 18269444 10.1111/j.1442-2042.2007.01953.x

[12] P. Zhou , X. Zhang , M. Guo , et al., “Ginsenoside Rb1 Ameliorates CKD‐Associated Vascular Calcification by Inhibiting the Wnt/β‐Catenin Pathway,” Journal of Cellular and Molecular Medicine 23 (2019): 7088–7098. 31423730 10.1111/jcmm.14611 PMC6787443

[13] M. Gao , T. Chen , L. Wu , X. Zhao , H. Mao , and C. Xing , “Effect of Pioglitazone on the Calcification of Rat Vascular Smooth Muscle Cells Through the Downregulation of the Wnt/β‐Catenin Signaling Pathway,” Molecular Medicine Reports 16 (2017): 6208–6213. 28849074 10.3892/mmr.2017.7308

[14] S. Reinhold , W. M. Blankesteijn , and S. Foulquier , “The Interplay of WNT and PPARγ Signaling in Vascular Calcification,” Cells 9 (2020): 2658. 33322009 10.3390/cells9122658 PMC7763279

[15] B. T. MacDonald , K. Tamai , and X. He , “Wnt/Beta‐Catenin Signaling: Components, Mechanisms, and Diseases,” Developmental Cell 17 (2009): 9–26. 19619488 10.1016/j.devcel.2009.06.016 PMC2861485

[16] S. J. Schunk , J. Floege , D. Fliser , and T. Speer , “WNT‐β‐Catenin Signalling—A Versatile Player in Kidney Injury and Repair,” Nature Reviews. Nephrology 17 (2021): 172–184. 32989282 10.1038/s41581-020-00343-w

[17] A. Vallée , J. N. Vallée , and Y. Lecarpentier , “Metabolic Reprogramming in Atherosclerosis: Opposed Interplay Between the Canonical WNT/β‐Catenin Pathway and PPARγ,” Journal of Molecular and Cellular Cardiology 133 (2019): 36–46. 31153873 10.1016/j.yjmcc.2019.05.024

[18] Y. Zhu , J. J. Ji , X. D. Wang , et al., “Periostin Promotes Arterial Calcification Through PPARγ‐Related Glucose Metabolism Reprogramming,” American Journal of Physiology. Heart and Circulatory Physiology 320 (2021): H2222–H2239. 33834866 10.1152/ajpheart.01009.2020

[19] E. Woldt , J. Terrand , M. Mlih , et al., “The Nuclear Hormone Receptor PPARγ Counteracts Vascular Calcification by Inhibiting Wnt5a Signalling in Vascular Smooth Muscle Cells,” Nature Communications 3 (2012): 1077. 10.1038/ncomms2087 PMC3523725 23011131

[20] G. D. Girnun , W. M. Smith , S. Drori , et al., “APC‐Dependent Suppression of Colon Carcinogenesis by PPARgamma,” Proceedings of the National Academy of Sciences of the United States of America 99 (2002): 13771–13776. 12370429 10.1073/pnas.162480299 PMC129773

[21] I. Takada , A. P. Kouzmenko , and S. Kato , “Wnt and PPARgamma Signaling in Osteoblastogenesis and Adipogenesis,” Nature Reviews Rheumatology 5 (2009): 442–447. 19581903 10.1038/nrrheum.2009.137

[22] Y. Xie , T. Lin , Y. Jin , et al., “Smooth Muscle Cell‐Specific Matrix Metalloproteinase 3 Deletion Reduces Osteogenic Transformation and Medial Artery Calcification,” Cardiovascular Research 120 (2024): 658–670. 38454645 10.1093/cvr/cvae035 PMC11074797

[23] N. Ding , Y. Lv , H. Su , et al., “Vascular Calcification in CKD: New Insights Into Its Mechanisms,” Journal of Cellular Physiology 238 (2023): 1160–1182. 37269534 10.1002/jcp.31021

[24] KDIGO , “KDIGO 2017 Clinical Practice Guideline Update for the Diagnosis, Evaluation, Prevention, and Treatment of Chronic Kidney Disease‐Mineral and Bone Disorder (CKD‐MBD),” Kidney International 7 (2017): 1–59. 10.1016/j.kisu.2017.04.001 PMC6340919 30675420

[25] T. Komori , “Regulation of Proliferation, Differentiation and Functions of Osteoblasts by Runx2,” International Journal of Molecular Sciences 20 (2019): 1694. 30987410 10.3390/ijms20071694 PMC6480215

[26] C. T. Chao , Y. P. Liu , S. F. Su , et al., “Circulating MicroRNA‐125b Predicts the Presence and Progression of Uremic Vascular Calcification,” Arteriosclerosis, Thrombosis, and Vascular Biology 37 (2017): 1402–1414. 28522697 10.1161/ATVBAHA.117.309566

[27] V. K. Rehan and J. S. Torday , “PPARγ Signaling Mediates the Evolution, Development, Homeostasis, and Repair of the Lung,” PPAR Research 2012 (2012): 289867. 22792087 10.1155/2012/289867 PMC3390135

[28] X. Wang , Y. Sun , J. Wong , and D. S. Conklin , “PPARγ Maintains ERBB2‐Positive Breast Cancer Stem Cells,” Oncogene 32 (2013): 5512–5521. 23770845 10.1038/onc.2013.217 PMC3898098

